# EDITAL DE SELEÇÃO

**Published:** 2024-07-29

**Authors:** Margareth Maria Pretti Dalcolmo, Marcia Margaret Menezes Pizzichini

**Affiliations:** 1 Presidente da SBPT; 2 Editora-Chefe do Jornal Brasileiro de Pneumologia

Brasília, 25 de junho de 2024

No período entre 01 de julho e 15 de setembro de 2024 estarão abertas as inscrições para candidatos ao cargo de Vice-Editor do Jornal Brasileiro de Pneumologia, com atuação no biênio 2025-2026. O Vice-Editor eleito assumirá a posição de Editor-Chefe em 2027, permanecendo na função por quatro anos (2027-2030) e, no biênio seguinte (2031-2032), assume novamente a função de Vice-Editor para auxiliar o novo Editor-Chefe.

Os interessados ao posto deverão ter experiência prévia como editor de periódicos de circulação internacional e enviar à administração da SBPT, em Brasília, suas propostas de gestão e Curriculum Vitae na plataforma Lattes.

As propostas dos candidatos deverão abranger os campos administrativo, científico e orçamentário, e deverão ser apresentadas em relação ao período de dois anos como Vice-Editor e aos quatro anos previstos para o futuro mandato como Editor-Chefe.

Os candidatos deverão conhecer as normas relativas à seleção do Vice-Editor e o funcionamento do Jornal Brasileiro de Pneumologia, descritas em seu regulamento, que pode ser obtido por meio de contato com a secretaria do JBP em Brasília.

